# Analysis of water resources carrying capacity and obstacle factors in Gansu section of the Wei River basin using combined weighting TOPSIS model

**DOI:** 10.1038/s41598-025-96828-4

**Published:** 2025-04-14

**Authors:** Kelong Duan, Zuirong Niu, Liang Cui

**Affiliations:** 1 College of Water Conservancy and Hydropower Engineering, Gansu Agricultural University, No. 1 Yingmen Village, Anning district, Lanzhou, 730070 Gansu China; 2 Hydrology and Water Resources Center of the Gansu Province, Lanzhou, 730000 Gansu China

**Keywords:** Water resources carrying capacity, Status standards, Combination weights, TOPSIS model, Obstacle factor, Hydrology, Environmental impact, Environmental impact

## Abstract

Water resource carrying capacity is an important indicator for measuring sustainable development. Given the rapid economic and social development in China today, coordinating the sustainable development of water resources, socio-economy, and eco-environment has become an urgent problem to be solved. This study takes the Gansu section of Wei River mainstream basin (GWRB) as a case study and constructs a three-dimensional WRCC evaluation system and status standards. Based on this research framework, we analyzed the trends in WRCC changes of GWRB from 2008 to 2022. Additionally, we conducted an in-depth study of the internal relationships and influencing factors within the WRCC system. The results show that the combination weighting method of multi-weight models avoids the one-sidedness of single weighting, leading to a more realistic distribution of weights. The result status standard derived from the indicator status standard prevents a disconnect between the result and the status, making the evaluation more rational and accurate. The WRCC of the GWRB increased from 0.098 (overloaded) in 2008 to 0.621 (weakly bearable) in 2022. During this period, the eco-environmental system improved from critical to bearable, while the socio-economic system improved from overloaded to weakly bearable. Due to geographical and climatic limitations, the water resource system continued to bear significant pressure and remained in overloaded state for most of the time. The key factors limiting the further improvement of WRCC in the GWRB are per capita water resources, utilization rate of water resources, COD emission per 10,000 yuan of GDP, ecological water use rate, water consumption per 10,000 GDP and agricultural water use rate. To improve the WRCC, we propose a series of targeted recommendations based on the research findings. The proposed research framework can also serve as a reference for related studies in arid and semi-arid regions.

## Introduction

Water resources are core elements of both the ecological environment and the socio-economic system. They are fundamental natural and strategic economic resources, playing a crucial role in social development^1^. The study of water resource carrying capacity (WRCC) is a rational evaluation of water resources and a scientific basis for optimizing water resource distribution, which aims to resolving the mismatch between regional water resources and the process of social development^2^. According to the United Nations, over the past century, human water consumption has increased sixfold. Due to factors such as population growth, changes in economic and consumption patterns, the global freshwater usage has been increasing at a rate of nearly 1% per year^3^. Currently, 50% of the world’s population still faces water scarcity^4^. For developing countries, inadequate water resource planning and management often worsen the situation, making this situation even more complex and severe^5^. Particularly in China, the high degree of economic and social development has dramatically increased the demand for water, causing increasing pressure on water resources^6^. Many regions have already exceeded the limit of WRCC, and water issues are becoming increasingly prominent, making it impossible to maintain sustainable economic and social development^7^. Moreover, issues such as excessive groundwater extraction and water pollution not only damage the ecosystem but also severely affect the normal lives of residents^8,9^. Therefore, it is generally believed that for a given region, the intensity and scale of its social development should be within the carrying capacity of its resource and environmental systems^10^. As a specific extension of the resource and environmental science domain, the WRCC is widely applied to understand the contradiction between limited water resources and social development. Exploring the internal connections of the WRCC system can alleviate water shortage and promote sustainable social development.

Up to now, research on WRCC has been continuously deepening and expanding both domestically and internationally. In terms of quantitative research, the methods have evolved from the past static analysis using a single indicator to the current dynamic comprehensive analysis using multiple indicators. Researchers evaluate WRCC by constructing a multi-dimensional evaluation index system and employing various methods within the weighted multi-criteria decision analysis model^11,12^. Among many evaluation methods, TOPSIS, as a typical representative of multi-criteria decision analysis models, is widely used in WRCC research due to its high flexibility and strong adaptability. However, its calculation process heavily relies on the weights of the indicators, which imposes strict requirements on the weighting models. Weighting models can be classified into two categories: subjective and objective. Subjective weighting models, such as Analytic Hierarchy Process (AHP)^13^ and the Delphi method^14^, allocate weights based on the decision-maker’s experience, which allows for the reflection of their preferences. However, the importance of indicators may vary across different decision-makers, leading to inconsistent results. Objective weighting models, such as the Entropy Weight Method (EWM)^15^, Criteria Importance Though Intercriteria Correlation(CRITIC)^16^, are entirely based on mathematical calculations of indicator data. These models can avoid the biases and errors inherent in subjective weighting methods, but they may also result in weights that contradict the actual importance of the indicators. Each method has its own advantages and limitations. Optimizing the combination of subjective and objective weight information can address the shortcomings of a single weighting model, resulting in a unified weighting outcome that integrates both subjective and objective perspectives^17^. For example, Xu et al.^18^ calculated the indicator weights by combining the entropy weighting method and the CRITIC method, and then used the GRA-TOPSIS evaluation method to assess the WRCC of Anhui Province. Zhu et al.^19^ combined three weighting models, AHP-EWM-COV, and utilized the TOPSIS model to analyze the regional variations in the WRCC among 31 provinces in China. Therefore, this study combines the AHP method and the CRITIC method to determine the indicator weights, as these two methods complement each other and overcome their individual limitations.

Although numerous studies provide different perspectives on WRCC research, the use of model-based quantitative evaluation can only present the trend changes of WRCC values without clearly indicating whether the WRCC exceeds the threshold in a given region^20^. To address this issue, relevant scholars have begun to establish a status standard for the results as a basis for determining whether the WRCC is within the acceptable range, quantifying the carrying capacity of WRCC based on the range in which the results fall. Chi et al.^21^ divided the WRCC scale from 0 to 1 into five intervals: [0, 0.6], [0.6, 0.69], [0.7, 0.79], [0.8, 0.89], and [0.9, 1], with each interval corresponding to a different water resource carrying capacity state. The interval in which the result falls determines whether the current water resource management policy should be maintained or revised. However, since the WRCC indicator system does not currently have a unified definition, different scholars may select varying indicators^22^. Thus, even when the same evaluation model is applied to the same region, the outcomes may differ due to variations in the indicator system. Therefore, this classification standard lacks universal applicability.

In summary, the existing studies have greatly enriched the content of WRCC, but some problems still need to be further explored. (1) The WRCC results lack a unified reference standard. Existing mathematical models can effectively quantify WRCC values, but without a reference standard, it is only possible to observe changes in the magnitude of WRCC. However, the specific changes in carrying capacity states still require further investigation. (2) Currently, there is no clear definition of the WRCC indicator system, nor is there any indicator that clearly explains the relationship between WRCC and socio-economic development. However, related indicators provide valuable information, which has led to an excess of WRCC indicators, with some overlapping in the information they provide.

**Fig. 1:**
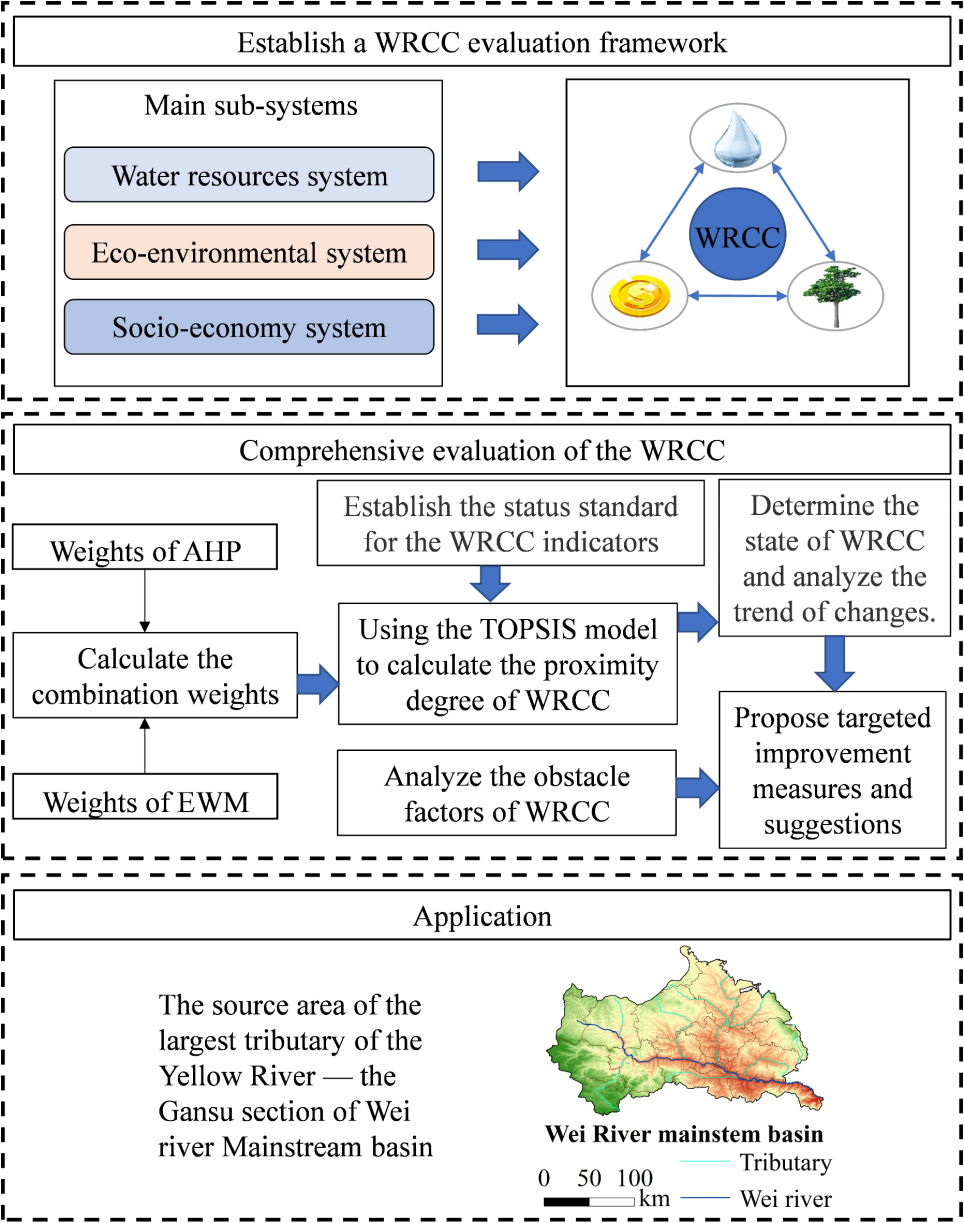
Research framework of this study. Note: The figure is generated by PowerPoint (https://www.microsoft.com/zh-cn/microsoft-365/powerpoint). The GWRB map is generated by ArcGIS 10.8 (https://desktop.arcgis.com/zh-cn/desktop/index.html), and so are the following maps. The map data is downloaded from DataV.GeoAtlas (https://datav.aliyun.com/portal/school/atlas/area_selector). The approval number is GS Jing (2022) 1061, and the boundary of the base map is not modified.

Based on the above issues, the main purpose of this study is to reasonably and qualitatively describe the state of WRCC on the basis of quantifying WRCC, with the aim of providing a comprehensive and thorough evaluation of the WRCC. Firstly, a comprehensive evaluation index system and status standards for water resources are established based on the water resource characteristics of the GWRB and considering China’s strictest water resource management system and relevant regional policies. To enhance the accuracy between the state description and the results, a series of grading standards are set for each indicator, and their critical values are incorporated into the calculation matrix. This approach allows for both the results and their corresponding status standards to be obtained simultaneously, thus avoiding any disconnect between the two. Secondly, a TOPSIS model with the weight distribution method combining the AHP and the CRITIC methods is used to calculate the WRCC and state thresholds for the GWRB from 2008 to 2022. Finally, an obstacle degree model is employed to identify the main factors hindering the improvement of WRCC, and targeted recommendations are provided for optimizing the water usage structure in the GWRB. The main contributions are as follows: (1) The use of the proposed indicator status standards to calculate the state thresholds of the results makes them more aligned with the actual conditions of the study area, thus providing a clear understanding of the WRCC state. (2) Based on the water resource characteristics of the GWRB, a water resource carrying capacity evaluation index system has been constructed, and the factors hindering further improvement of the WRCC have been analyzed. The research results can provide decision-making bases for the comprehensive development and utilization of water resources in the Wei River Basin in the future, assist in sustainable development within the basin, and provide scientific bases for administrative departments to improve water-saving policy standards. At the same time, it can provide reference for the evaluation of WRCC in other arid and semi-arid regions of China. The structure of the paper is as follows: Section 2 provides an overview of the study area, research methods, and data sources. Section 3 presents an analysis of the research results. Section 4 discusses the findings. Section 5 summarizes the conclusions of the study. The specific research framework is shown in Fig. 1.

## Study area and data

### Overview of the study area

**Fig. 2:**
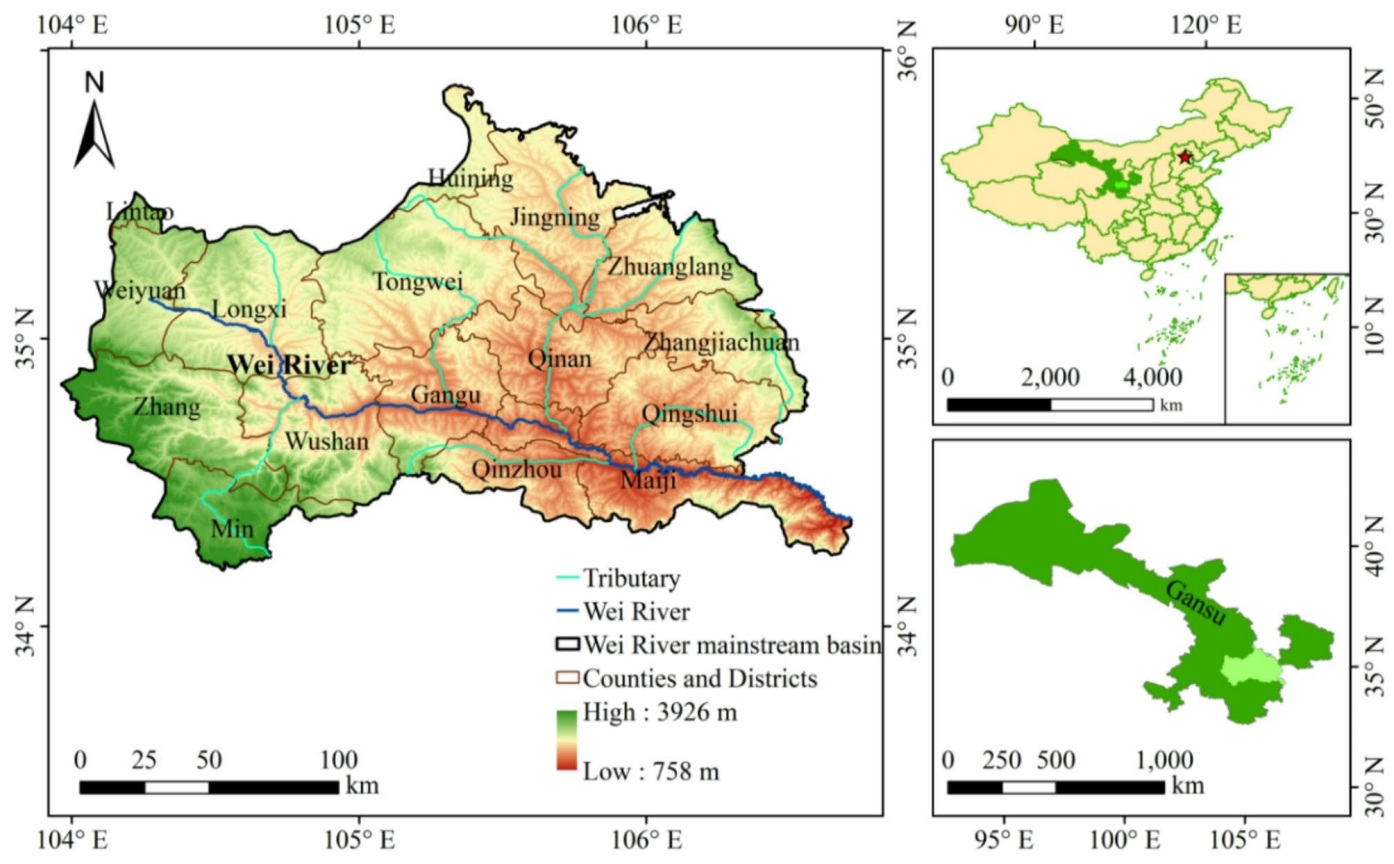
Schematic diagram of the GWRB.

As the largest tributary of the Yellow River, the Wei River holds an important position in the governance and development of the Yellow River basin, and is equally significant in the national strategy of the Western Development^23^. However, the Wei River itself suffers from insufficient water resources and limited WRCC^24^. With the development of industrial and agricultural production, the Wei River is facing issues such as water scarcity, severe supply-demand conflicts, and serious water pollution^25^. The health and vitality of the Wei River are under serious threat. Due to the large number of tributaries and the basin covers Gansu and Shaanxi provinces, it is generally not studied as a whole. The study area selected for this study is the Gansu section of the Wei River mainstem basin (GWRB, Fig. 2). The Gansu section of the Wei River starts at Weiyuan County in Dingxi City and ends at the junction of Maiji District in Tianshui City and Baoji City in Shaanxi Province. The basin area is 2.58 × 10^4^ km², and the river length is approximately 360 km^26^. In 2022, the total water resources in the GWRB were 11.17 × 10^8^ m³, the permanent population was 443 × 10^4^ people, and the GDP was 1221 × 10^8^ yuan. However, the per capita water resources were only 252 m³/person, less than 1/3 of the national per capita water resources. With only 4.8% of the water resources in Gansu Province, the GWRB held 17.8% of the population and 10.9% of the GDP. Given the rapid and concentrated development of population and industries, economic activities in the GWRB are constantly increasing, and the WRCC is facing a severe challenge.

### Data sources

The data used in this paper are from the Gansu Water Resources Bulletin and the Gansu Statistical Yearbook. With 2008–2022 as the evaluation period, 15 representative indicators were selected, and the raw data for the 15 years were obtained by organizing and analyzing the relevant data in the bulletins and yearbooks. The corresponding indicator data were calculated from these raw data. Among them, data on ammonia nitrogen, COD, and riverine effluent discharges are missing for 2020–2022. For the missing data, interpolation based on the adjacent four years data is used to fill in the gaps.

### Research methods

#### Establishment and grading of the indicators system

Establishing an indicator system is a prerequisite for evaluating the WRCC. Existing studies show that significant differences exist across regions in terms of social development, economic structure, water resources endowment, and ecological environment^27^. Therefore, evaluation indicators should be selected based on the specific conditions of the study area to better reflect its regional characteristics^28^. This study, based on a thorough analysis of the water resources endowment, socio-economic development, and ecological environment in the GWRB, selects 15 core indicators from both “quantity” and “quality” perspectives that accurately reflect the WRCC of GWRB. The WRCC indicator system constructed in this study is divided into three hierarchical layers. The target layer represents WRCC, reflecting the overall allocation of water resources. The criterion layer consists of the water resources system, the eco-environmental system, and the socio-economic system. The indicator level reflects the specific indicators of the three subsystems and serves as the operational foundation of the entire comprehensive evaluation system. The constructed WRCC evaluation system of GWRB is shown in Table 1. “-” represents the negative benefit index. The larger the negative benefit index, the smaller the WRCC. The “+” represents the positive benefit indicator. The larger the positive benefit indicator, the larger the WRCC.

**Table 1:** Comprehensive evaluation index system of WRCC.

Target layer	Criterion layer	Indicator layer	Calculation method or source	Indicator unit	Attribute
WRCC(A)	Water resources system(B1)	Per capita water resources (C1)	Total water resources/ Total population	m³/people	+
		Utilization rate of water resources (C2)	Total water use / Total water resources·100%	%	−
		Modulus of water production (C3)	Total water resources / Area	10^4^ m³/km^2^	+
		Modules of water supply (C4)	Total water supply / Area	10^4^ m³/km^2^	+
		Annual precipitation (C5)	*Gansu Province Water Resources Bulletin*	×10^8^ m³	+
	Eco-environmental system(B2)	Wastewater discharge of per 10,000 yuan GDP (C6)	Wastewater discharge / The gross product of basin	t/10^4^ yuan	−
		Ammonia and nitrogen emissions per 10,000 yuan of GDP (C7)	Ammonia emissions / The gross product of basin	kg/10^4^ yuan	−
		COD emission per 10,000 yuan of GDP (C8)	COD emissions / The gross product of basin	kg/10^4^ yuan	−
		Ratio of sewage volume to runoff (C9)	Volume of sewage entering the river / Runoff volume	–	−
		Water consumption rate (C10)	Total water consumption / Total water use	%	−
		Ecological water use rate (C11)	Total ecological water use / Total water use	%	+
	Socio−economic system(B3)	Water consumption per 10,000 GDP (C12)	Total water use / The gross product of basin	m³/10^4^ yuan	−
		Industrial water use rate (C13)	Total industrial water use / Total water use	%	−
		Agricultural water use rate (C14)	Total water use in agriculture / Total water use	%	−
		Per capita daily domestic water consumption (C15)	Total residential water consumption/ Total population /365	m³/people·d^− 1^	−

The WRCC is divided into five levels of status, with reference to the Gansu Province Industrial Water Quota (2023 Edition) and the existing WRCC evaluation standards^29–31^. Level I means that the WRCC has reached the standard of sustainable development, and maintaining this water use structure will ensure that the WRCC remains balanced over the long term. Level II indicates that the WRCC is able to carry the impacts of human activities on the water resources system, but the carrying capacity is weak and cannot be sustained for an extended period. Without intervention the water resources system will collapse. Level III indicates that the WRCC is barely within its carrying capacity and is on the verge of being overloaded. Level IV indicates that the WRCC has been overloaded; at this time, the water resources system is under significant pressure and timely changes to the water structure are required. Level V indicates that the WRCC is seriously overloaded, and the water resources system can no longer support the long-term development of society. All indicators are associated with the five status levels above and are classified according to their respective threshold values. The specific indicator thresholds for the WRCC status standards are shown in Table 2.

**Table 2:** WRCC indicator status level thresholds.

Evaluation indicators	Level I Bearable	Level II Weakly bearable	Level III Critical	Level IV Overloaded	Level V Severely overloaded
C1	> 2100	[2100,1700)	[1700,1100)	[1100.500]	< 500
C2	< 20	[20,40)	[40,60)	[60,80]	> 80
C3	> 60	[60,35)	[35,20)	[20,10]	< 10
C4	> 20	[20,15)	[15,10)	[10,5]	< 5
C5	> 800	[800,600)	[600,400)	[400,200]	< 200
C6	< 10	[10,25)	[25,40)	[40,55]	> 55
C7	< 0.1	[0.1,0.3)	[0.3,0.5)	[0.5,1]	> 1
C8	< 1	[1,3)	[3,5)	[5,10]	> 10
C9	< 1	[1,2)	[2,5)	[5,10]	> 10
C10	< 40	[40,55)	[55,70)	[70,85]	> 85
C11	> 6	[6,3)	[3,1)	[1,0.5]	< 0.5
C12	< 24	[24,60)	[60,120)	[120,180]	> 180
C13	< 10	[10,15)	[15,20)	[20,25]	> 25
C14	< 40	[40,45)	[45,50)	[50,60]	> 60
C15	< 0.06	[0.06,0.07)	[0.07,0.08)	[0.08,0.1]	> 0.1

#### Weighting models

The indicator items and their weights in the WRCC indicator system are the core of this research framework. Since the indicator threshold values and data together form the decision matrix, the scientific validity and accuracy of indicator weights directly affect the reliability of the results and the validity of the status standards. Some indicators may not fully reflect their importance in terms of numerical values, but in practice, they may play a critical role^32^. The AHP method leverages expert knowledge and experience to help us accurately assess the relative importance of these indicators. Furthermore, for some indicators whose importance cannot be solely determined by subjective judgment, objective methods are essential. The CRITIC method objectively calculates weights by analyzing the contrast and conflict between indicators, effectively reducing redundant information and improving the rationality of weight distribution^33^. Considering the differences in the computational foundations of the two methods, this study combines AHP and CRITIC methods to utilize the strengths of both. Ultimately, comprehensive weight values are derived through the multiplication aggregation method. This combined approach not only improves the scientific rigor of weight determination but also better reflects the actual impact of each indicator in WRCC evaluation. The specific calculation steps of the AHP method are described in the literature^34^. The specific calculation steps of the CRITIC method are shown in the literature^35^.

#### TOPSIS method

The WRCC system is essentially a multi-objective decision-making system, comprising subsystems from multiple dimensions^36^. The core challenge in conducting a comprehensive evaluation of WRCC lies in effectively incorporating each indicator into the decision-making framework and performing a calculation to assess WRCC^37^. The TOPSIS model, due to its ability to consider multiple evaluation indicators comprehensively, allows for horizontal comparisons within the same year as well as vertical comparisons across different years^38^. This enables it to accurately reveal the spatial distribution and temporal variation patterns of WRCC, making it particularly suitable for evaluating carrying capacity in regions with water scarcity. The main calculation steps of the method^39^ are as follows:

1. After the negative indicators are homogenized, the matrix is normalized according to Eq. (1) to obtain the normalized decision matrix $V={({v_{ij}})_{m \times n}}$.
  1 $${v_{ij}}=\frac{{{x_{ij}}}}{{\sqrt {\sum\limits_{{i=1}}^{m} {x_{{ij}}^{2}} } }}$$
  where *x*_*ij*_ denotes the value of the j-th indicator in year i.
2. Constructing the weighting matrix
  2 $$Z={({z_{ij}})_{m \times n}}$$
  Where${z_{ij}}={w_{ij}} \times {v_{ij}}$, *w*_*ij*_ is the weight of the j-th indicator of the i-th criterion layer.
3. Determination of positive and negative ideal solutions for each indicator
  3 $$z_{j}^{ + } = \max \{ z_{{1j}} ,z_{{2j}} ,...,z_{{mj}} \} (j = 1,2,...,n)$$
  4 $$z_{j}^{ - } = \min \{ z_{{1j}} ,z_{{_{2} j}} ,...,z_{{_{m} j}} \} (j = 1,2,...,n)$$
4. Express the distance of each evaluation object from the optimal solution in terms of Euclidean distance
  5 $$D_{i}^{+}=\sqrt {\sum\limits_{{j=1}}^{n} {{{[({z_{ij}}-z_{j}^{+})]}^2}} }$$
  6 $$D_{i}^{ - }=\sqrt {\sum\limits_{{j=1}}^{n} {{{[({z_{ij}}-z_{j}^{-})]}^2}} }$$
  The larger the value of D^+^, the further away the evaluation object is from the optimal solution; the larger the value of D^−^, the further away the evaluation object is from the worst solution.
5. Calculate the proximity degree of WRCC
  7 $${C_i}=\frac{{D_{i}^{ - }}}{{D_{i}^{+}+D_{i}^{ - }}}$$
  Where 0 ≤ *C*_*i*_≤1, the closer *C*_*i*_ is to 1, indicating that the evaluation object is closer to the ideal program, the better the evaluation results, and vice versa, the worse.

#### Obstacle degree model

WRCC analysis involves not only measuring how well water resources meet the needs of the economy, society, and the environment, but also identifying weaknesses in WRCC. Identifying the main obstacles to WRCC allows for targeted recommendations to be made for regional development. The main computational steps of the obstacle degree model^40^ are as follows:

1. Calculate the factor contribution *F*_*ij*_, the indicator deviation *I*_*ij*_
  8 $${F_{{\text{ij}}}}={W_{ij}} \times {W_i}$$
  9 $${I_{{\text{ij}}}}=1 - {R_{ij}}$$
  where *W*_*i*_ is the weight of the criterion layer to which the j-th indicator belongs, *R*_*ij*_ is the normalized indicator value.
2. Calculation of the degree of impediment of indicator j to the carrying capacity of water resources *P*_*j*_
  10 $${P_j}=\frac{{({F_j} \times {I_j})}}{{\sum\limits_{{j=1}}^{n} {{F_j} \times {I_j}} }} \times 100\%$$

## Analysis of results

### Results of weighting calculations

The AHP was used for subjective weighting, CR = 0.068 < 0.1, and the consistency test proved that the assignment is reasonable. The CRITIC method was used to calculate the objective weights, and two weighting results were obtained, from which the combination weights were calculated. The results of the calculated weights are shown in Table 3.

**Table 3:** Evaluation indicator weights of WRCC.

Target Layer	Criterion layer	Indicator layer	AHP weight	CRITIC weight	Combined weight
WRCC(A)	Water resources system(B1)	Per capita water resources (C1)	0.0738	0.0514	0.0597
		Utilization rate of water resources (C2)	0.1555	0.0531	0.1300
		Modulus of water production (C3)	0.0274	0.0567	0.0244
		Modules of water supply (C4)	0.0199	0.0872	0.0273
		Annual precipitation (C5)	0.0512	0.0617	0.0498
	Eco-environmental system(B2)	Wastewater discharge of per 10,000 yuan GDP (C6)	0.1019	0.0596	0.0955
		Ammonia and nitrogen emissions per 10,000 yuan of GDP (C7)	0.0478	0.0550	0.0414
		COD emission per 10,000 yuan of GDP (C8)	0.0478	0.0570	0.0429
		Ratio of sewage volume to runoff (C9)	0.027	0.0708	0.0301
		Water consumption rate (C10)	0.0164	0.0572	0.0148
		Ecological water use rate (C11)	0.1703	0.0836	0.2242
	Socio-economic system(B3)	Water consumption per 10,000 GDP (C12)	0.1659	0.0540	0.1409
		Industrial water use rate (C13)	0.0293	0.0711	0.0328
		Agricultural water use rate (C14)	0.0498	0.0758	0.0594
		Per capita daily domestic water consumption (C15)	0.0161	0.1056	0.0268

### Results of the WRCC evaluation

A standardized decision matrix was formed by combining the critical values of the indicator state with the values of the evaluation indicator, and the WRCC of GWRB from 2008 to 2022 was evaluated using TOPSIS model based on combined weights. The evaluation results and status grading criteria are shown in Table 4.

**Table 4:** Comprehensive evaluation results of WRCC.

Year	WRCC	Water resources system	Eco-environmental system	Socio-economic system
2022	0.621	0.119	0.806	0.472
2021	0.558	0.338	0.662	0.457
2020	0.489	0.514	0.504	0.398
2019	0.547	0.227	0.709	0.283
2018	0.337	0.435	0.317	0.317
2017	0.217	0.132	0.219	0.271
2016	0.212	0.010	0.201	0.331
2015	0.192	0.099	0.197	0.238
2014	0.140	0.100	0.140	0.168
2013	0.228	0.426	0.156	0.171
2012	0.171	0.234	0.161	0.128
2011	0.122	0.143	0.122	0.103
2010	0.101	0.077	0.112	0.077
2009	0.085	0.036	0.099	0.065
2008	0.098	0.104	0.105	0.070
I	0.627	0.871	0.508	0.849
II	0.314	0.528	0.229	0.306
III	0.146	0.320	0.057	0.093
IV	0.052	0.120	0.016	0.023

### Analysis of WRCC results

**Fig. 3:**
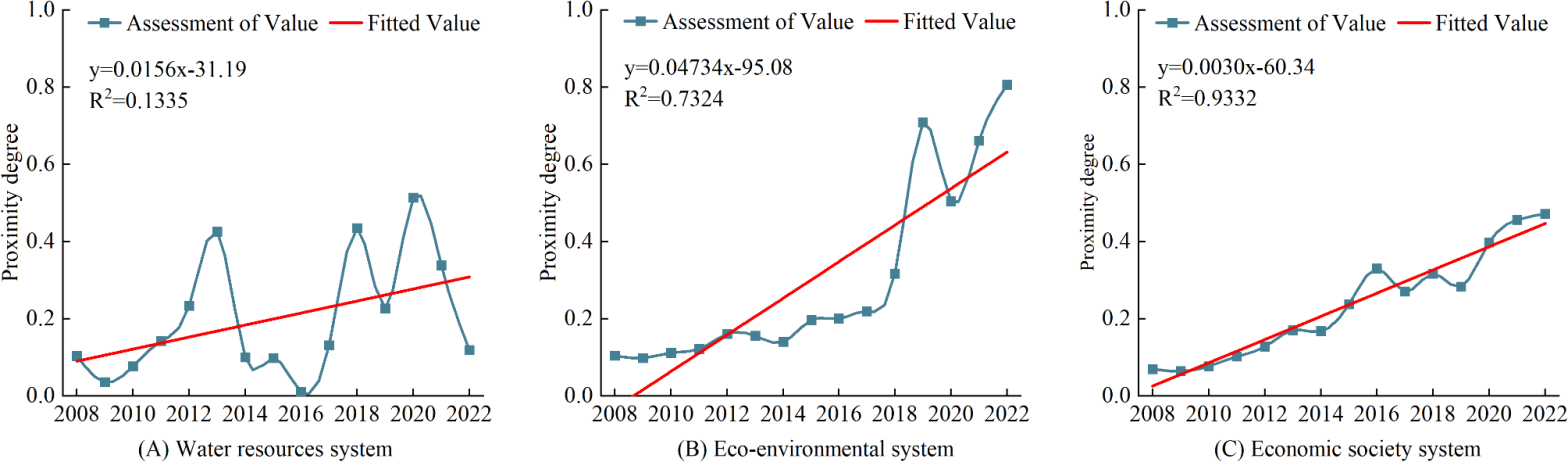
Subsystem evaluation results of WRCC.

**Fig. 4:**
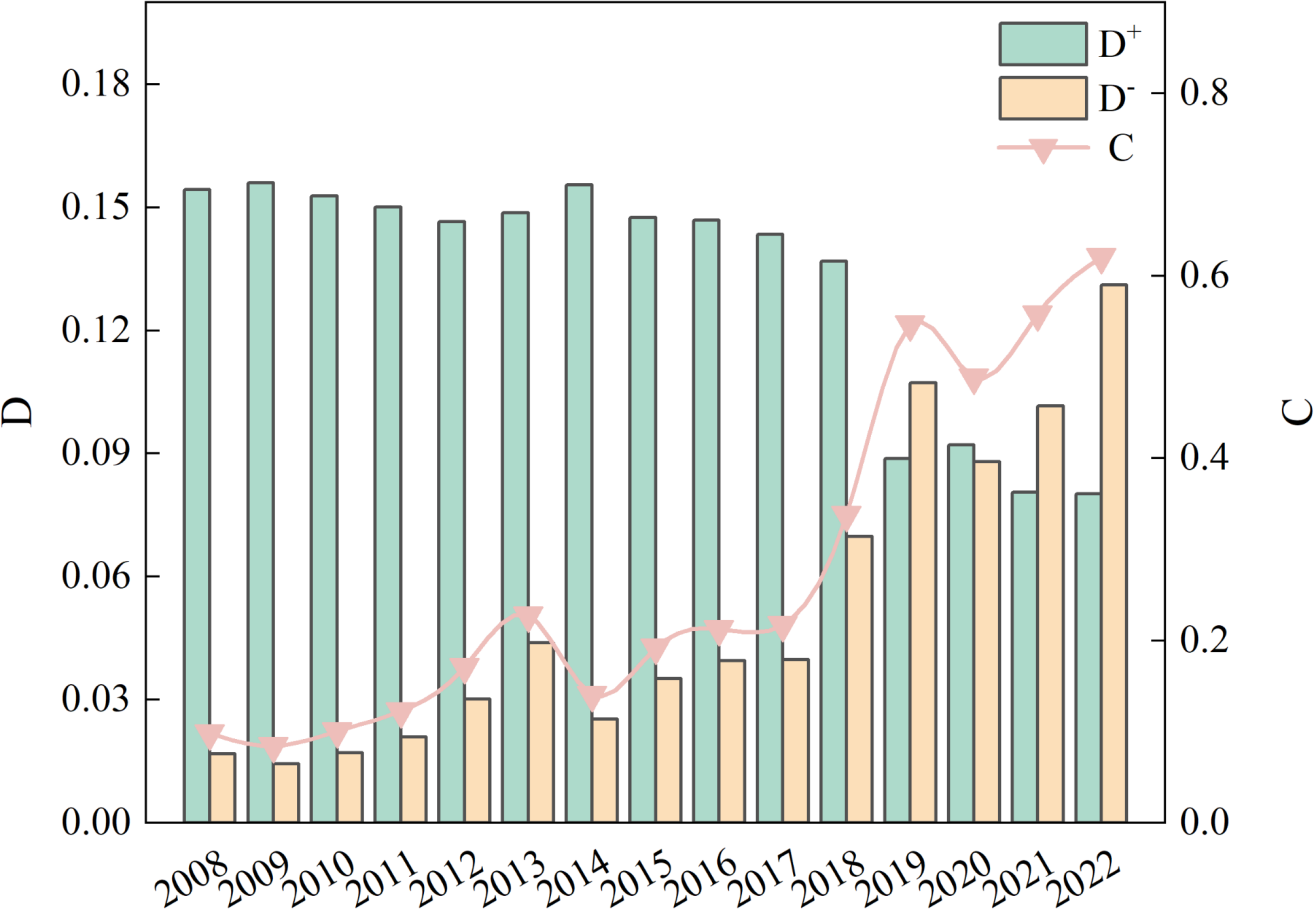
Comprehensive evaluation results of WRCC.

Based on Table 4, the change curve of WRCC of the GWRB was drawn, as shown in Figs. 3 and 4. The WRCC of GWRB is analyzed below.

1. Analysis of the water resources system
  The results of the water resources system evaluation (Fig. 3A) show that the carrying capacity of the water resources system has fluctuated dramatically over the research period, with a weak upward trend of “W”. The carrying capacity of the water resources system transitioned from level V to level III from 2008 to 2013, decreased to level V from 2014 to 2016, then fluctuated upward, reaching to level IV in 2017 and 2019, to level III in 2018, 2020 and 2021, and finally decreased to level V in 2022. After an in-depth analysis of the internal causes in the context of the actual situation, it was found that the fluctuation of the system is mainly caused by changes in per capita water resources, utilization rate of water resources, and annual precipitation, which are directly or indirectly related to the total water resources of the GWRB. The total water resources in the GWRB are mainly replenished by rainfall. As a natural phenomenon, rainfall exhibits significant instability in terms of frequency, intensity, and spatial distribution, which leads to huge inter-annual variations in the total water resources, thus causing fluctuations in the indicators of per capita water resources and utilization rate of water resources. The interannual variation range of per capita water resources in the GWRB from 2008 to 2022 is 138.1–647.9 m³/ person, with a total variation of 509.821 m³/person. The inter-annual variation range of water resources exploitation and utilization rate is 16.71-75.99%, with a total distance of 59.28%. The limited amount of water resources in the GWRB, the high rate of water consumption and the increasing water demand, a variety of factors intertwined with each other to make the pressure on the water resources system continue to climb.
2. Analysis of the eco-environmental system.
  Figure 3B shows the evaluation results of the eco-environmental system. The eco-environmental system carrying capacity rose steadily from 2008 to 2018, transitioning from level III to level II. The eco-environmental system carrying capacity increased exponentially from 2018 to 2022, reaching a maximum value of 0.806 (level I) in 2022. The increase in the carrying capacity of the ecological environment system is due to the decrease in wastewater discharge of per 10,000 yuan GDP and the increase in the ecological water use rate. In 2008, Shaanxi Province recognized that the ecology of the GWRB was under serious threat and carried out a five-year key management of the GWRB. In 2011, it cooperated with the cities of Tianshui and Dingxi to establish an ecological compensation mechanism for the entire GWRB. Subsequently, efforts focused on the development and utilization of water resources and protection continued to enhance the integrated ecological management of the GWRB, promoting the upstream pollution control, ecological protection of water sources, and introducing several important policies. Over the years, the wastewater discharge of per 10,000 yuan GDP has decreased significantly, and in 2022, the wastewater discharge of per 10,000 yuan GDP was reduced to 5.82 t / 10,000 yuan, which is only 25.92% of that in 2008. Guaranteeing ecological water use plays a significant role in promoting the recuperation of water ecosystems, curbing the trend of water ecological degradation, and enhancing the function and stability of river and lake ecosystems. In 2008, the ecological water consumption of the GWRB was 0.008 billion m³, and the ecological water use rate was 1.5%, while by 2022, the ecological water consumption was 0.057 billion m³, and the ecological water use rate had increased to 10.78%. Continuous improvement of ecological environment quality in the GWRB is a crucial initiative to promote economic and social development in harmony with the carrying capacity of water resources.
3. Analysis of socio-economic system.
  Figure 3C shows that the socio-economic carrying capacity level of the GWRB has steadily increased from 2008 to 2022, with the evaluation value rising from 0.07 (level IV) in 2008 to 0.472 (level II) in 2022. The results show a good trend of stable and progressive economic development in the GWRB, which clearly indicates that the economic strength of various administrative regions in the GWRB has continuously increased. The per capita daily domestic water consumption in the GWRB has remained relatively stable and has not significantly affected changes in the socio-economic systems. The GWRB’s industrial structure is mainly based on agriculture, with industry playing a supplementary role. Agricultural water use has been consistently high, which hinders the improvement of WRCC. However, industrial water use has been decreasing year by year, alleviating the pressure on water resources. The water consumption per 10,000 GDP during the research period decreased sharply from 177.7 m³/10,000 yuan in 2008 to 43.4 m³/10,000 yuan in 2022, which was already lower than the national average of that year. It has greatly alleviated the water pressure brought by economic and social development. Over the years, the administrative regions in the GWRB have regarded water resources as the biggest rigid constraint. They have promoted a shift in water use from extensive to intensive, focusing on water conservation and improving water efficiency, while supporting the steady, green, and high-quality development of the economy and society with limited water resources.
4. Overall analysis of WRCC.
  Figure 4 shows that the GWRB’s comprehensive WRCC evaluation value has increased from 0.098 to 0.621, and the carrying capacity has been upgraded from Level IV to Level II, showing an overall upward trend. However, there are some fluctuations within the research period, such as decreased evaluation value in 2014 and 2020. The main reason for the decline in the WRCC evaluation value in 2014 was the lowest ecological water use rate of 0.62% and the highest agricultural water use rate of 70.14%. The main reason for the decrease in WRCC in 2020 was the reduction in the modules of water supply to 1.87 × 10^4^ m³/km^2^, which was the lowest during the research period. D^−^ increased from 0.017 in 2008 to 0.131 in 2022, while D^+^ decreased from 0.154 in 2008 to 0.080 in 2022, indicating that the WRCC is gradually moving away from the negative ideal solution and towards the positive ideal solution. According to the WRCC evaluation, the research period can be roughly divided into two stages. The first stage (2008–2017) was the preliminary improvement stage. The WRCC was mainly influenced by eco-environmental and socio-economic systems, reaching a critical state by the end of this phase. The second stage (2018–2022) was the significant improvement stage. Through sustained efforts to improve the water ecological environment, promoted the green transformation of industries, and advanced major water conservancy projects, the carrying capacity of the eco-environmental and socio-economic systems has been further enhanced. Although the water resources system remains unstable, it has improved significantly compared to the first stage. The evaluation results show that the development of eco-environment and socio-economy in the GWRB can be coordinated at present, but the unstable water resources system is currently the main factor hindering further improvement of WRCC.

### Obstacle degree analysis of WRCC

**Fig. 5:**
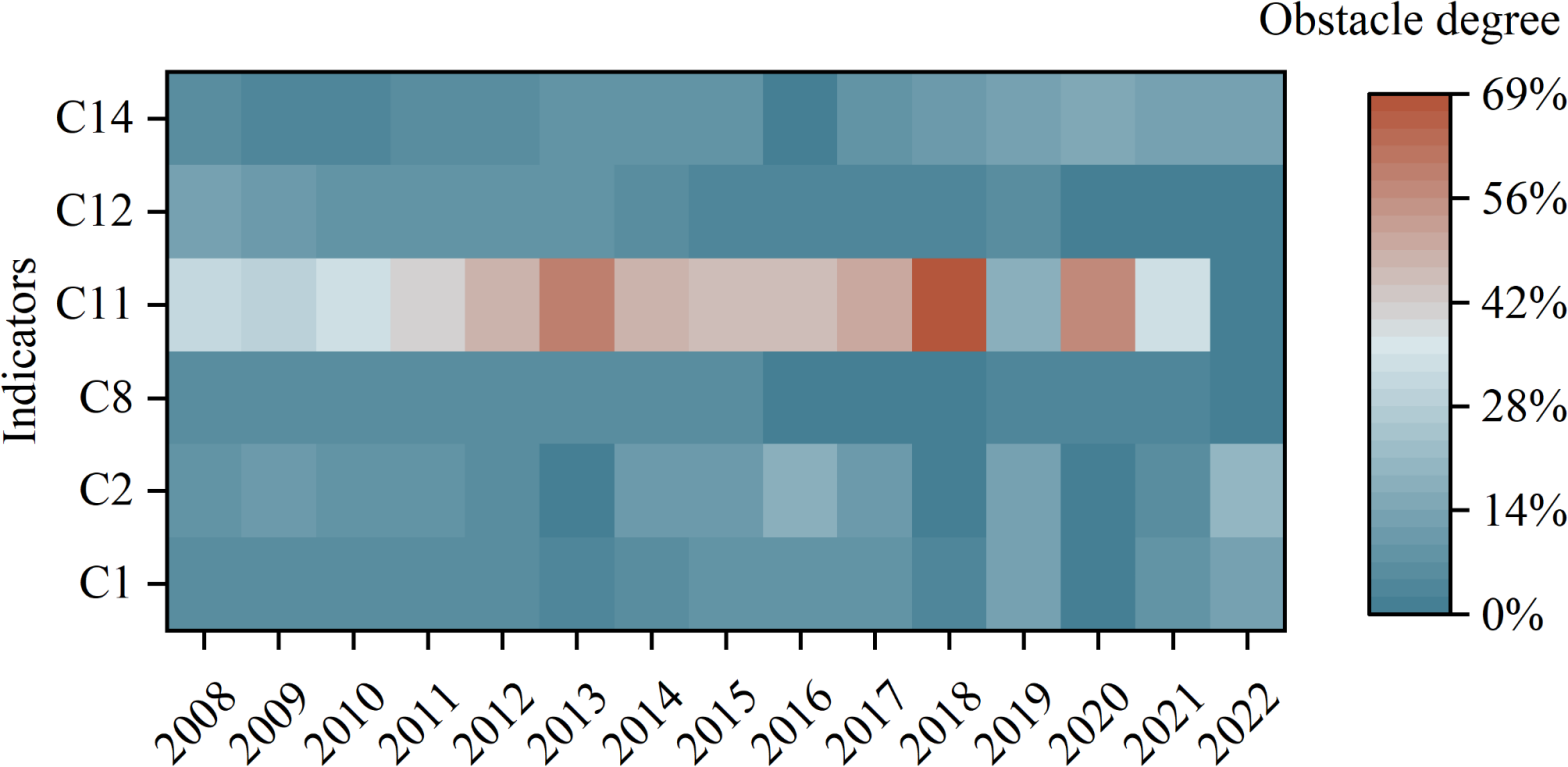
Heat map of the main obstacle factors of WRCC.

The obstacle degree model was used to calculate the obstacle degree of the WRCC index in the GWRB from 2008 to 2022. The six main obstacle factors were selected based on the criteria of obstacle degree *P*_*j*_≥5.0% and occurrence frequency greater than 50%, as shown in Fig. 5.

Figure 5 illustrates that the obstacles to per capita water resources, utilization rate of water resources, and agricultural water use rate have been increasing year by year, indicating that the demand for water resources in the GWRB is increasing. However, the amount of local water resources is insufficient to support the supply and demand requirements. Agriculture is overly dependent on irrigation, and natural rainfall is scarce, so water resources are exploited more intensively and deeply. The obstacle degree of COD emission per 10,000 yuan of GDP and Water consumption per 10,000 GDP decreases year by year, which indicates that the water saving and emission reduction work in the GWRB has achieved remarkable results. Along with economic growth, water resource consumption is decreasing, which is a crucial way to achieve water resource conservation and efficient utilization. Ecological environmental water use was consistently low before 2019, which did not match the rapid development needs of the GWRB, resulting in an increasing obstacle degree of ecological water use rate year by year. After 2020, efforts to improve the ecological environment of GWRB have been intensified, and a number of ecological protection, restoration and construction projects have been implemented, vigorously promoting the construction of ecological civilization. From then on, the obstacle degree of ecological water consumption rate began to decrease, and the negative impact on the carrying capacity of water resources gradually decreased.

From the classification of obstacle factors, there are two main obstacle factors in each sub-system, which verifies that the WRCC is a composite system in which water resources, eco-environmental and socio-economy are closely coupled. The indicators within the system affect each other.

## Discussion

### Discussion on the rationality of the evaluation results of WRCC

As the differences in the selection of evaluation models and indicators can lead to deviations in the final evaluation results, it is necessary to analyze the rationality of the results of this study.

As the core of the calculation, the allocation of indicator weights directly affects the rationality and validity of the results. Zhang et al.^41^ calculated the indicator weights using the EWM and employed the TOPSIS model to evaluate the WRCC of the Gansu section of the Yellow River Basin based on a four-dimensional WRCC indicator system. Their results showed that from 2012 to 2021, the average WRCC of the Longmen-Sanmenxia region was 0.144, with only a slight improvement over the years. It is slightly different from the results of this study. There are two main reasons for this discrepancy. Firstly, the previous study used only the entropy weight method to calculate the weights, leading to an uneven distribution of indicator weights. Among the 13 indicators selected in the previous study, three indicators, namely per capita water resources, water production modulus, and ecological water use rate, accounted for more than 50% of the total weight, which caused the evaluation results to be overly dependent on the values of these three indicators. Secondly, the difference in the research scales is another important factor. The Longmen-Sanmenxia region covers the Wei River, Jing River, and Beiluo River basins in Gansu, making its study area broader than the area selected in this study. The principle of the generalized substitutability of resources and the near-far interaction relationship demonstrate that a shortage of one resource can be compensated by the availability of superior resources nearby^42^. This implies that the WRCC of GWRB is influenced by surrounding regions, leading to a deviation in WRCC compared to the Longmen-Sanmenxia region. Wang et al.^43^ used the AHP and EWM methods to calculate indicator weights and evaluate the WRCC in Gansu. Their results showed that the WRCC fluctuated between 0.366 and 0.785 from 2008 to 2020, with the carrying capacity status improving from an overloaded state to a weakly bearable state. Although there are numerical differences between their study and the present one, the descriptions of the WRCC status are highly consistent. The results of the present study indicate that the WRCC status in the GWRB has been continuously improving, with an average WRCC of 0.274. Notably, a significant improvement occurred after 2017, which is most closely related to the ecological environment system. This notable improvement is mainly attributed to the comprehensive implementation of the 13th Five-Year Plan for ecological and environmental protection, which effectively promoted ecological conservation and contributed to a significant enhancement of the ecological system carrying capacity. By 2022, the WRCC had risen to a weakly bearable state.

### Discussion on the WRCC status standards

Indicator status standards, as important benchmarks for assessing and comparing the quality and status of WRCC, play a crucial role in determining whether WRCC meets the requirements for sustainable development. The application of these standards provides a solid basis for the rational allocation of water resources and the formulation of scientific water resource management policies. However, most current WRCC studies have not established unified indicator status standards^44^. Instead, they tend to divide the results into several intervals to quantify the WRCC status. For example, Deng et al.^45^ used the TOPSIS model to evaluate the WRCC of the Dongting Lake Basin from 2009 to 2018 and classified it into five levels. Zhang et al.^46^. based on the distribution of results, employed the natural break method to categorize the WRCC into five levels. While such simple classification methods may offer preliminary judgments in some cases, they are inadequate to fully reflect the actual state of WRCC as a complex dynamic system. This study, based on local water resource characteristics and socio-economic development, established more scientific threshold values for WRCC status and used four proximity degree (C) critical values to refine the classification of WRCC status. This approach makes the evaluation of WRCC status more objective and reasonable. However, this study still relies on previous research and the authors’ personal understanding when setting the classification thresholds. Therefore, the standard has certain limitations. Future research should further clarify the scientific definition of water resources carrying capacity and its evaluation standards in order to provide more comprehensive and accurate decision-making support for water resource management.

## Conclusions and policy implications

In order to accurately evaluate the WRCC of the GWRB, a three-dimensional WRCC evaluation index system based on the water resources characteristics was constructed to evaluate the WRCC of GWRB from 2008 to 2022. The conclusions reached are as follows:

1. The TOPSIS model based on combined weights for comprehensive evaluation avoids the one-sidedness of single weights and makes the evaluation results more realistic and accurate. The results show that the WRCC of the GWRB increased from 0.098 in 2008 to 0.621 in 2022. The state of WRCC had shifted from overloaded to weakly bearable. The overall carrying capacity of water resources is increasing, but there is still a lot of room for development and potential for improvement.
2. By analyzing the internal relationships of the WRCC system, it was found that the pressure on the GWRB water resource system has not significantly improved, which may be closely related to the region’s water resource endowment characteristics. GWRB is located in an arid and semi-arid climate region, where water resources are scarce and mainly rely on precipitation for replenishment, leading to a naturally weaker WRCC. Although some engineering measures, such as water diversion projects, can alleviate this issue to some extent, constructing water diversion projects is a large-scale and time-consuming undertaking that cannot provide sufficient water resources to the region in the short term. Therefore, the GWRB should primarily focus on improving water use efficiency and enhancing water-saving benefits to strengthen WRCC.
3. The results of the obstacle degree model indicate that the main obstacle factors to the WRCC of the GWRB are per capita water resources, utilization rate of water resources, COD emission per 10,000 yuan of GDP, ecological water use rate, water consumption per 10,000 GDP and agricultural water use rate. In this regard, the GWRB will need to implement improvement measures for these indicators in the future, addressing the conflicts between the water resources system, the economic and social system, and the eco-environmental system. Efforts should be made to further optimize water resource allocation, refine the process of water use, promote water conservation policies, reduce pollution discharge, properly replenish water to the ecological environment, gradually eliminate environmental risks. These measures will ultimately enhance the water resource carrying capacity of the GWRB.

## Data Availability

The datasets used and/or analysed during the current study available from the corresponding author on reasonable request.

## References

[1] Zhang, J. B. et al. Multiperspective-driven factorial metabolic network analysis framework for energy-water nexus vulnerability assessment and management-policy simulation. *J. Environ. Manage.* **315**, 115095. 10.1016/j.jenvman.2022.115095 (2022). 35525039 10.1016/j.jenvman.2022.115095

[2] Peng, T., Deng, H. W., Lin, Y. & Jin, Z. Y. Assessment on water resources carrying capacity in karst areas by using an innovative DPESBRM concept model and cloud model. *Sci. Total Environ.* **767**, 144353. 10.1016/j.scitotenv.2020.144353 (2021). 33434832 10.1016/j.scitotenv.2020.144353

[3] UESCO. The United Nations World Water Development Report. *United Nations*. (2024). 10.18356/9789213589113. (2024).

[4] Zhang, F., Wu, Z., Di, D. & Wang, H. Water resources allocation based on water resources supply-demand forecast and comprehensive values of water resources. *J. Hydrol. Reg. Stud.* **47**, 101421. 10.1016/j.ejrh.2023.101421 (2023).

[5] Jing, P. et al. Spatiotemporal evolution of sustainable utilization of water resources in the Yangtze river economic belt based on an integrated water ecological footprint model. *J. Clean. Prod.* **358**, 132035. 10.1016/j.jclepro.2022.132035 (2022).

[6] Dong, H. et al. Uncovering regional disparity of China’s water footprint and inter-provincial virtual water flows. *Sci. Total Environ.* **500**, 120–130. 10.1016/j.scitotenv.2014.08.094 (2014). 25222751 10.1016/j.scitotenv.2014.08.094

[7] Ji, J. T., Qu, X. N., Zhang, Q. & Tao, J. Predictive analysis of water resource carrying capacity based on system dynamics and improved fuzzy comprehensive evaluation method in Henan Province. *Environ. Monit. Assess.* **194**(7), 500. 10.1007/s10661-022-10131-7 (2022). 35701693 10.1007/s10661-022-10131-7

[8] Jing, P. et al. Coupling coordination and Spatiotemporal dynamic evolution of the water-energy-food-land (WEFL) nexus in the Yangtze river economic belt, China. *Environ. Sci. Pollut Res.* **30**(12), 34978–34995. 10.1007/s11356-022-24659-1 (2023). 10.1007/s11356-022-24659-1 36525198

[9] Chen, S. et al. Comprehensive assessment of water environmental carrying capacity for sustainable watershed development. *J. Environ. Manage.* **303**, 114065. 10.1016/j.jenvman.2021.114065 (2022). 34823905 10.1016/j.jenvman.2021.114065

[10] Wang, X., Zhang, S., Tang, X. & Gao, C. Spatiotemporal heterogeneity and driving mechanisms of water resources carrying capacity for sustainable development of Guangdong Province in China. *J. Clean. Prod.* **412**, 137398. 10.1016/j.jclepro.2023.137398 (2023).

[11] Lv, B. et al. Evaluation of the water resource carrying capacity in Heilongjiang, Eastern China, based on the improved TOPSIS model. *Ecol. Indic.* **150**, 110208. 10.1016/j.ecolind.2023.110208 (2023).

[12] Zhang, J. T. & Dong, Z. C. Assessment of coupling coordination degree and water resources carrying capacity of Hebei Province (China) based on WRESP2D2P framework and GTWR approach. *Sustain. Cities Soc.* **82**, 103862. 10.1016/j.scs.2022.103862 (2022).

[13] Bu, J. H., Li, C. H., Wang, X., Zhang, Y. & Yang, Z. W. Assessment and prediction of the water ecological carrying capacity in Changzhou City, China. *J. Clean. Prod.* **277**, 123988. 10.1016/j.jclepro.2020.123988 (2020).

[14] Jung, Y. & Choi, M. Survey-based approach for hydrological vulnerability indicators due to climate change: Case study of Small-Scale rivers. *J. Am. Water Resour. Assoc.* **48**(2), 256–265. 10.1111/j.1752-1688.2011.00608.x (2012).

[15] Song, Q. R., Wang, Z. C. & Wu, T. H. Risk analysis and assessment of water resource carrying capacity based on weighted Gray model with improved entropy weighting method in the central plains region of China. *Ecol. Indic.* **160**, 111907. 10.1016/j.ecolind.2024.111907 (2024).

[16] Zhang, Y. M. et al. Assessment of agricultural water resources carrying capacity and analysis of its spatio-temporal variation in Henan Province, China. *J. Clean. Prod.* **403**, 136869. 10.1016/j.jclepro.2023.136869 (2023).

[17] Li, Q. S., Liu, Z. H., Yang, Y. H., Han, Y. & Wang, X. P. Evaluation of water resources carrying capacity in Tarim river basin under game theory combination weights. *Ecol. Indic.* **154**, 110609. 10.1016/j.ecolind.2023.110609 (2023).

[18] Xu, W. et al. Evaluation and analysis of spatio-temporal variation of water resources carrying capacity and restraining factor: A case study in Anhui Province, China. *Mitig Adapt. Strat Gl* **29**(5), 50. 10.1007/s11027-024-10143-3 (2024).

[19] Zhu, Q. Y. & Cao, Y. Research on provincial water resources carrying capacity and coordinated development in China based on combined weighting TOPSIS model. *Sci. Rep.* **14**(1), 12497. 10.1038/s41598-024-63119-3 (2024). 38822005 10.1038/s41598-024-63119-3 PMC11143342

[20] Peng, T. & Deng, H. Comprehensive evaluation on water resource carrying capacity based on DPESBR framework: A case study in Guiyang, Southwest China. *J. Clean. Prod.* **268**, 122235. 10.1016/j.jclepro.2020.122235 (2020).

[21] Chi, M. et al. Evaluation of water resources carrying capacity in ecologically fragile mining areas under the influence of underground reservoirs in coal mines. *J. Clean. Prod.* **379**, 134449. 10.1016/j.jclepro.2022.134449 (2022).

[22] Wang, X., Zhang, S., Gao, C. & Tang, X. Coupling coordination and driving mechanisms of water resources carrying capacity under the dynamic interaction of the water-social-economic-ecological environment system. *Sci. Total Environ.* **920**, 171011. 10.1016/j.scitotenv.2024.171011 (2024). 38369138 10.1016/j.scitotenv.2024.171011

[23] Gao, X. et al. Evaluating river health through respirogram metrics: insights from the Weihe river basin, China. *Sci. Total Environ.* **919**, 170805 (2024). 38342463 10.1016/j.scitotenv.2024.170805

[24] Zhou, Y. et al. The study on Spatial distribution of water ecological environment carrying capacity during extreme drought conditions. *Sci. Rep.* **14**(1), 11986. 10.1038/s41598-024-62856-9 (2024). 38796635 10.1038/s41598-024-62856-9 PMC11128009

[25] Mao, R. et al. Evaluating multifaceted effects of watershed properties and human activities on drought propagation in the Wei river basin with an integrated framework. *Sci. Total Environ.* **926**, 171712. 10.1016/j.scitotenv.2024.171712 (2024). 38494024 10.1016/j.scitotenv.2024.171712

[26] Li, Y. et al. How to price ecosystem water yield service and determine the amount of Compensation?—The Wei river basin in China as an example. *Land* **11**(7), 1118–1118 (2022).

[27] Lu, L., Lei, Y., Wu, T. & Chen, K. Evaluating water resources carrying capacity: The empirical analysis of Hubei Province, China 2008–2020. *Ecol. Indic.* **144**, 109454. 10.1016/j.ecolind.2022.109454 (2022).

[28] Chen, Q. Y., Zhu, M. T., Zhang, C. J. & Zhou, Q. The driving effect of spatial-temporal difference of water resources carrying capacity in the yellow river basin. *J. Clean. Prod.* **388**, 135709. 10.1016/j.jclepro.2022.135709 (2023).

[29] Mou, S. Y. et al. A comprehensive evaluation model of regional water resource carrying capacity: Model development and a case study in Baoding, China. *Water* **12**(9), 2637. 10.3390/w12092637 (2020).

[30] Wu, Y. et al. Assessment of water resources carrying capacity based on fuzzy comprehensive evaluation - case study of Jinan, China. *Water Supply* **21**(2), 513–524. 10.2166/ws.2020.335 (2021).

[31] Zhang, Y., Song, X. M., Wang, X. J., Jin, Z. F. & Chen, F. Multi-Level fuzzy comprehensive evaluation for water resources carrying capacity in Xuzhou City. *China Sustain.* **15**(14), 11369. 10.3390/su151411369 (2023).

[32] Yang, Z. et al. Comprehensive evaluation and scenario simulation for the water resources carrying capacity in Xi’an City, China. *J. Environ. Manage.* **230**, 221–233. 10.1016/j.jenvman.2018.09.085 (2019). 30290309 10.1016/j.jenvman.2018.09.085

[33] Zhao, S. et al. Ecological risk assessment and spatial-temporal differentiation of soil and water resources in the Hefei metropolitan area. *Sci. Rep.* **14**(1), 8462. 10.1038/s41598-024-59246-6 (2024). 38605176 10.1038/s41598-024-59246-6 PMC11009403

[34] Lee, S. Determination of priority weights under multiattribute decision-making situations: AHP versus fuzzy AHP. *J. Constr. Eng. M*. **141**(2), 05014015. 10.1061/(asce)co.1943-7862.0000897 (2015).

[35] Shi, H. T., Li, Y. F., Jiang, Z. N. & Zhang, J. Comprehensive power quality evaluation method of microgrid with dynamic weighting based on CRITIC. *Meas. Control* **54**(5–6), 1097–1104. 10.1177/00202940211016092 (2021).

[36] Yan, L., Jiao, D. & Zhan, Y. Evaluation of regional water resources carrying capacity in China based on variable weight model and grey-markov model: a case study of Anhui Province. *Sci. Rep.* **13**(1). 10.1038/s41598-023-40487-w (2023). 10.1038/s41598-023-40487-w PMC10439155 37596286

[37] Zhu, Q. & Cao, Y. Research on provincial water resources carrying capacity and coordinated development in China based on combined weighting TOPSIS model. *Sci. Rep.* **14**(1), 12497. 10.1038/s41598-024-63119-3 (2024). 38822005 10.1038/s41598-024-63119-3 PMC11143342

[38] Zhang, J. X. et al. Safety resilience evaluation of hydrogen refueling stations based on improved TOPSIS approach. *Int. J. Hydrogen Energy*. **66**, 396–405. 10.1016/j.ijhydene.2024.04.129 (2024).

[39] Behzadian, M., Otaghsara, S. K., Yazdani, M. & Ignatius, J. A state-of the-art survey of TOPSIS applications. *Expert Syst. Appl.* **39**(17), 13051–13069. 10.1016/j.eswa.2012.05.056 (2012).

[40] Xu, X. Y., Zhang, Z. H., Long, T., Sun, S. M. & Gao, J. Mega-city region sustainability assessment and Obstacles identi Fi cation with GIS-entropy-TOPSIS model: A case in Yangtze river delta urban agglomeration, China. *J. Clean. Prod.* **294**, 126147. 10.1016/j.jclepro.2021.126147 (2021).

[41] Zhang, F., Yin, M. & Zhang, J. Comprehensive evaluation of water resources carrying capacity in Gansu section of the yellow river basin based on entropy-weight-TOPSIS model. *Yellow River* **46**(04), 79–85. 10.3969/j.issn.1000-1379.2024.04.013 (2024). (In Chinese).

[42] Xu, M. & Bao, C. Elastic range measurement of resource and environmental carrying capacity and future scenario analysis: A case study of the Lanzhou-Xining urban agglomeration. *Resour. Sci.* **45**(10), 1961–1976 (2023). (In Chinese).

[43] Wang, J., Yang, Y. & Sheng, Q. Evaluation of water resources carrying capacity in Gansu Province based on improved TOPSIS model. *Water Resour. Power* **40**(11), 35–39. 10.20040/j.cnki.1000-7709.2022.20220149 (2022). (In Chinese).

[44] Wang, X., Liu, L., Zhang, S. & Gao, C. Dynamic simulation and comprehensive evaluation of the water resources carrying capacity in Guangzhou City, China. *Ecol. Indic.* **135**, 108528. 10.1016/j.ecolind.2021.108528 (2022).

[45] Deng, Z. H., Dai, L. Q., Deng, B. & Tian, X. Y. Evaluation and spatial-temporal evolution of water resources carrying capacity in Dongting lake basin. *J. Water Clim. Change* **12**(5), 2125–2135. 10.2166/wcc.2021.210 (2021).

[46] Zhang, X. & Duan, X. Evaluating water resource carrying capacity in Pearl river-West river economic belt based on portfolio weights and GRA-TOPSIS-CCDM. *Ecol. Indic.* **161**, 111942. 10.1016/j.ecolind.2024.111942 (2024).

